# An AI-based multiphase framework for improving the mechanical ventilation availability in emergency departments during respiratory disease seasons: a case study

**DOI:** 10.1186/s12245-024-00626-0

**Published:** 2024-04-02

**Authors:** Miguel Ortiz-Barrios, Antonella Petrillo, Sebastián Arias-Fonseca, Sally McClean, Fabio de Felice, Chris Nugent, Sheyla-Ariany Uribe-López

**Affiliations:** 1 Centro de Investigación en Gestión e Ingeniería de Producción (CIGIP), Universitat Politecnica de Valencia, Camino de Vera, s/n, Valencia, 46022 Spain; 2 Department of Productivity and Innovation, Universidad de la Costa CUC, Barranquilla, 080002 Colombia; 3 Department of Engineering, University of Naples “Parthenope”, Naples, Italy; 4 School of Computing, Ulster University, Belfast, BT15 1ED UK; 5 Academic Multidisciplinary Division of Jalpa de Mendez, Juarez Autonomous University of Tabasco, Jalpa de Mendez, Tabasco, Mexico

**Keywords:** Artificial Intelligence (AI), Random Forest (RF), Discrete-Event-Simulation (DES), Emergency Department (ED), Mechanical ventilation, Healthcare

## Abstract

**Background:**

Shortages of mechanical ventilation have become a constant problem in Emergency Departments (EDs), thereby affecting the timely deployment of medical interventions that counteract the severe health complications experienced during respiratory disease seasons. It is then necessary to count on agile and robust methodological approaches predicting the expected demand loads to EDs while supporting the timely allocation of ventilators. In this paper, we propose an integration of Artificial Intelligence (AI) and Discrete-event Simulation (DES) to design effective interventions ensuring the high availability of ventilators for patients needing these devices.

**Methods:**

First, we applied Random Forest (RF) to estimate the mechanical ventilation probability of respiratory-affected patients entering the emergency wards. Second, we introduced the RF predictions into a DES model to diagnose the response of EDs in terms of mechanical ventilator availability. Lately, we pretested two different interventions suggested by decision-makers to address the scarcity of this resource. A case study in a European hospital group was used to validate the proposed methodology.

**Results:**

The number of patients in the training cohort was 734, while the test group comprised 315. The sensitivity of the AI model was 93.08% (95% confidence interval, [88.46 − 96.26%]), whilst the specificity was 85.45% [77.45 − 91.45%]. On the other hand, the positive and negative predictive values were 91.62% (86.75 − 95.13%) and 87.85% (80.12 − 93.36%). Also, the Receiver Operator Characteristic (ROC) curve plot was 95.00% (89.25 − 100%). Finally, the median waiting time for mechanical ventilation was decreased by 17.48% after implementing a new resource capacity strategy.

**Conclusions:**

Combining AI and DES helps healthcare decision-makers to elucidate interventions shortening the waiting times for mechanical ventilators in EDs during respiratory disease epidemics and pandemics.

## Background

### The rising importance of mechanical ventilation availability in seasonal respiratory diseases

Since the onset of several respiratory viruses, including influenza, Respiratory Syncytial Virus (RSV), and the recent COVID-19, EDs everywhere have faced unpreceded challenges in managing respiratory infections in the community, protecting their staff and patients from infection, and managing resources, including the development of innovative new resources for prevention and treatment of these diseases, managing the associated supply chains, and ensuring efficient and equitable resource allocation. This problem has been particularly severe in countries with resource-limited health facilities [1].

The high demand, and often serious supply shortages, for severely respiratory-affected patients has been widespread, with the insufficient mechanical ventilator supply causing serious problems and mortalities in many countries [2]. Such demands emanated from large numbers of patients requiring ventilators for sustained use [2].

Throughout the respiratory disease seasons, epidemiological models have been widely used and disseminated to provide understanding, forecast the future spread of these diseases, and assess the likely impact of possible interventions. Such modeling can provide useful insights into forecasting resource requirements, such as mechanical ventilation demand. Complementary to these population models, simulation has emerged to improve understanding and support decision-making throughout the EDs, thus reducing the overall impact of respiratory viruses [3]. Simulation models allow us to incorporate complexities such as high heterogeneity of disease dynamics across patients alongside huge uncertainties of disease trajectories and patient behaviors [4]. For instance, ventilation of patients is highly variable because of the heterogeneous patient lung pathology [5]. Such simulation models can facilitate resource planning [6] alongside the epidemiological population models. This is in response to the mechanical ventilation needs evident in the recent respiratory pandemic, where a fatality rate ranging between 50% and 97% was experienced in patients needing mechanical ventilation [7, 8].

### Managing artificial ventilator availability: a review of efforts

#### Predicting the use of mechanical ventilators

Such crises have led to various models supporting ED surge capacity planning [9]. In this regard, we are initially required to predict patients with high artificial ventilator probability during the next few hours. This has been described by Parreco et al. [10] as a complex classification problem with related variables, known as *features*, and some AI algorithms that may be suitable for providing accurate predictions. Allied with this problem is the challenge of determining the probabilities of such invasive treatments. AI could be useful for early detection of patient deterioration, identification of new prognostic features, and management improvements during respiratory epidemics/pandemics. Many recent papers have used AI when addressing seasonal respiratory diseases. For example, Prodhan et al. [11] employed multiple regression models based on chest radiographs to predict the mechanical ventilation duration (> 8 days) in RSV-infected children. Morton et al. [12] used the P/F ratio to predict the artificial ventilator need during the UK influenza. Likewise, Parreco et al. [10] predicted prolonged mechanical ventilation and tracheostomy placement through gradient-boosted decision trees. Later, Patrício et al. [13] used various classifiers to predict COVID-19 patient admission to hospital and respiratory assistance requirements. Overall, most studies concluded that AI was suitable for identifying individuals who may require respiratory support in the future.

From the discussion above, we can conclude that it is possible to provide reasonable predictions for the progression of patients to artificial ventilators within EDs. Here, we are mainly interested in using the RF classifier, which is an ensemble method that combines multiple decision trees, so it is flexible and addresses heterogeneity well [14].

#### Reconfiguring the ED for improved mechanical ventilation availability

There have been numerous studies and reviews concerned with ED simulation modeling [15–17]. A key issue is the inclusion of patient-centered care pathways for long-term and complex patient management [18, 19], and management of scarce resources [20]. The heterogeneity and diversity of patient pathways are often modeled using multiple compartments [20]. Another common focus is the resource allocation within the ED and other healthcare services e.g., Ordu et al. [21], developed a novel healthcare resource allocation decision support tool that links all services and specialties within hospitals.

Moving to our current study on modeling mechanical ventilator availability, not surprisingly it transpires that several simulation models have already been developed. For example, Bhavani et al. [22] simulated different triage strategies for ventilator allocation to COVID-19 patients in an extreme ventilator shortage environment, facilitating a better understanding of ethical issues in such strategies. Scarce resource allocation of artificial ventilators to suitable patients has also been explored by Mehrotra et al. [23], where the authors considered stochastic optimization solutions that allocate ventilators from a central agency.

In this paper, we use a novel strategy that couples Artificial Intelligence (AI), where RF is applied to predict artificial ventilator use, with Discrete-Event Simulation (DES), where we model the patient pathway through ED, including the pretesting of strategies reducing the mechanical ventilation waiting time. This new approach facilitates the optimal resource utilization for such patients during respiratory disease seasons.

## Methods

### Study design, setting, and population

**Fig. 1:**
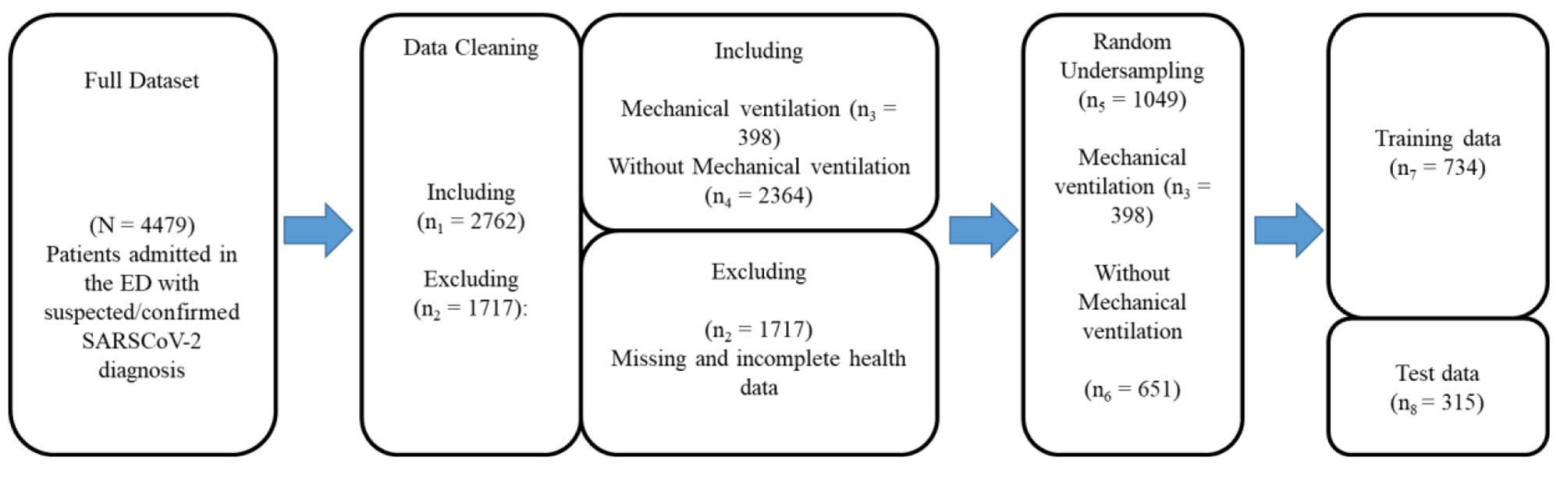
Flowchart representing the pathway from the initial patient dataset to the derivation of training and test cohorts

A cross-sectional scheme was implemented in the ED of a Spanish hospital group during the recent respiratory pandemic – SARS-CoV-2. The healthcare group provided full consent on this project through Agreement #14-12-2021-004 (Access request ID:39). In this ED, the Emergency Severity Index was employed to support the triage process [24] in 4,479 Covid-19-infected patients. Also, an internal mechanical ventilation section for patients needing this device was implemented. Despite this, prolonged waiting times for ventilators were reported, evidencing the need for better resource management. The data employed in this study were collated from the “COVID DATA SAVE LIVES” Electronic Health Records from February-2020 to February-2021. Patients with incomplete or missing backgrounds were excluded from this analysis. Specifically, 1,717 patients were discarded due to missing data, including potential predictors and outcomes (Fig. 1).

### Features and missing data management

The dataset derived from the Electronic Health Records (EHRs) of the showcased hospital contained 13 characteristics from the clinical (Systolic Arterial Pressure (SAP_R1, SAP_R2), Diastolic Arterial Pressure (DAP_R1, DAP_R2), Core Temperature (CT_R1, CT_R2), Oxygen Saturation Level (OSL_R1, OSL_R2), Heartbeat (HB_R1, HB_R2), D-dimmer concentration: DDIMER) and sociodemographic (Sex, Age) domains. The features’ significance was validated through an Analysis of Variance (ANOVA) test (α = 0.05) and the Mean Decrease in Gini Coefficient (MDGC). Chest radiographs were not utilized, considering availability limitations and interpretation variability. Also, respiratory rate and blood gas analysis features were not deemed as they were not available in the EHRs. Finally, the median values were computed to impute the missing data.

### Outcomes

We set a binary outcome variable for the mechanical ventilation use. Specifically, “1” was assigned to patients under assisted artificial ventilation; otherwise, “0” was allocated. Therefore, it is possible to estimate the probability of using this machine once the patient has been admitted to the ED. Thereby, ED managers can anticipate designing interventions to ensure high mechanical ventilation availability.

### Data processing and model formulation

As there is no independent cohort, we have split the dataset to train and assess the AI model (Fig. 1). The training group comprised 70% randomly chosen patients (${n}_{7}=734$) while the testing dataset contained 30% random cases (${n}_{8}=315$). The training subset was employed to train the classifier to predict the mechanical ventilation probability, whilst the testing subset was utilized to evaluate how well the RF model forecasts this likelihood.

### RF algorithm

An RF classifier was trained and assessed to verify how well it could score the mechanical ventilation probability in the ED. The model hyperparameters were tuned by implementing cross-validation. The number of trees varied between 100 and 500 while the number of variables tried in each split was 3. The Rstudio® (v. 2023.09.1 + 494) and R® (v. 4.3.2) were employed to operationalize this algorithm.

### DES for mechanical ventilation capacity management

A DES model was designed to mimic the current ED from admission to artificial ventilation provision. We first diagrammed the multi-phase process in the ED using a flowchart. The process variable data were then gathered, cleaned, and analyzed regarding randomness, homogeneity, and goodness-of-fit. The DES model was later constructed in Arena® (v. 16.10.00) to animate the patient routes within the ED, thereby easing commitment with the stakeholders and supporting model verification. A 1-sample sign test (α = 0.05) was employed to validate whether the real-world ED was statistically comparable with its virtual version concerning mechanical ventilation waiting time. The RF predictions were inserted into the simulated system to appraise whether the artificial ventilator availability is sufficient to address the incoming demand. Finally, various improvement scenarios are designed and pre-tested in the simulation model. Man-Whitney tests (α = 0.05) are performed to validate if the median waiting time for artificial ventilation will be further reduced if implementing the proposed intervention.

## Results

A total of 1,049 respiratory-affected patients were considered in this analysis. Table 1 elucidates the characteristics of patients who required/did not require ventilation support. For example, most patients who required ventilation support were male (*n* = 292; 0.73); while a large proportion of the ventilated patients were over 60 years (*n* = 288; 0.72). Regarding clinical variables, the median OSL was lower in ventilated patients (OSL_1 = 92%; OLS_2 = 92%) than in those who did not necessitate this treatment (OSL_1 = 95%; OSL_2 = 95%). Furthermore, the median number of heartbeats was more significant in mechanically ventilated people (HB_1 = 91; HB_2 = 91 Vs. HB_1 = 88; HB_2 = 88). Moreover, it is worth noting the substantial difference between the medians of D-Dimmer levels detected in patients interacting with this device (D-DIMER = 1334 ng/ml) and those who did not (D-DIMER = 745 ng/ml).

**Table 1:** Characteristics of patients who required/did not require ventilation support

Feature	Levels	Patients who required ventilation support	Patients who did not require ventilation support	*P*-value
*Sex*	*Male*	292 (0.73)	373 (0.57)	< 0.01
	*Female*	106 (0.27)	278 (0.43)	
*Age*	*30 years old or younger*	14 (0.04)	17 (0.03)	< 0.05
	*30–60 years old*	96 (0.24)	200 (0.31)	
	*Older than 60*	288 (0.72)	434 (0.66)	
*Systolic Arterial Pressure (mm Hg)*	*Record 1 (SAP_1)*	130.00 (234.85)	133.00 (353.26)	< 0.005
	*Record 2 (SAP_2)*	131.00 (270.57)	133.00 (358.14)	
*Diastolic Arterial Pressure (mm Hg)*	*Record 1 (DAP_1)*	76.00 (90.252)	76.00 (886.70)	< 0.01
	*Record 2 (DAP_2)*	76.00 (116.087)	77.00 (705.28)	
*Core Temperature (°C)*	*Record 1 (CT_1)*	36.7 (0.527)	36.5 (0.466)	< 0.005
	*Record 2 (CT_2)*	36.7 (0.571)	36.5 (4.698)	
*Oxygen Saturation Level (%)*	*Record 1 (OSL_1)*	92.00 (73.664)	95.00 (29.791)	< 0.005
	*Record 2 (OSL_2)*	92.00 (92.224)	95.00 (29.413)	
*Heartbeat* *(# of heartbeats)*	*Record 1 (HB_1)*	91.00 (205.155)	88.00 (211.362)	< 0.01
	*Record 2 (HB_2)*	91.00 (235.223)	88.00 (214.949)	
*D-dimmer concentration (ng/ml)*	--------	1334 (54,071,055)	745 (13,318,254)	< 0.001

*Note: In categorical variables: Frequency (Proportion) // In quantitative variables: Median (Variance)

**Fig. 2:**
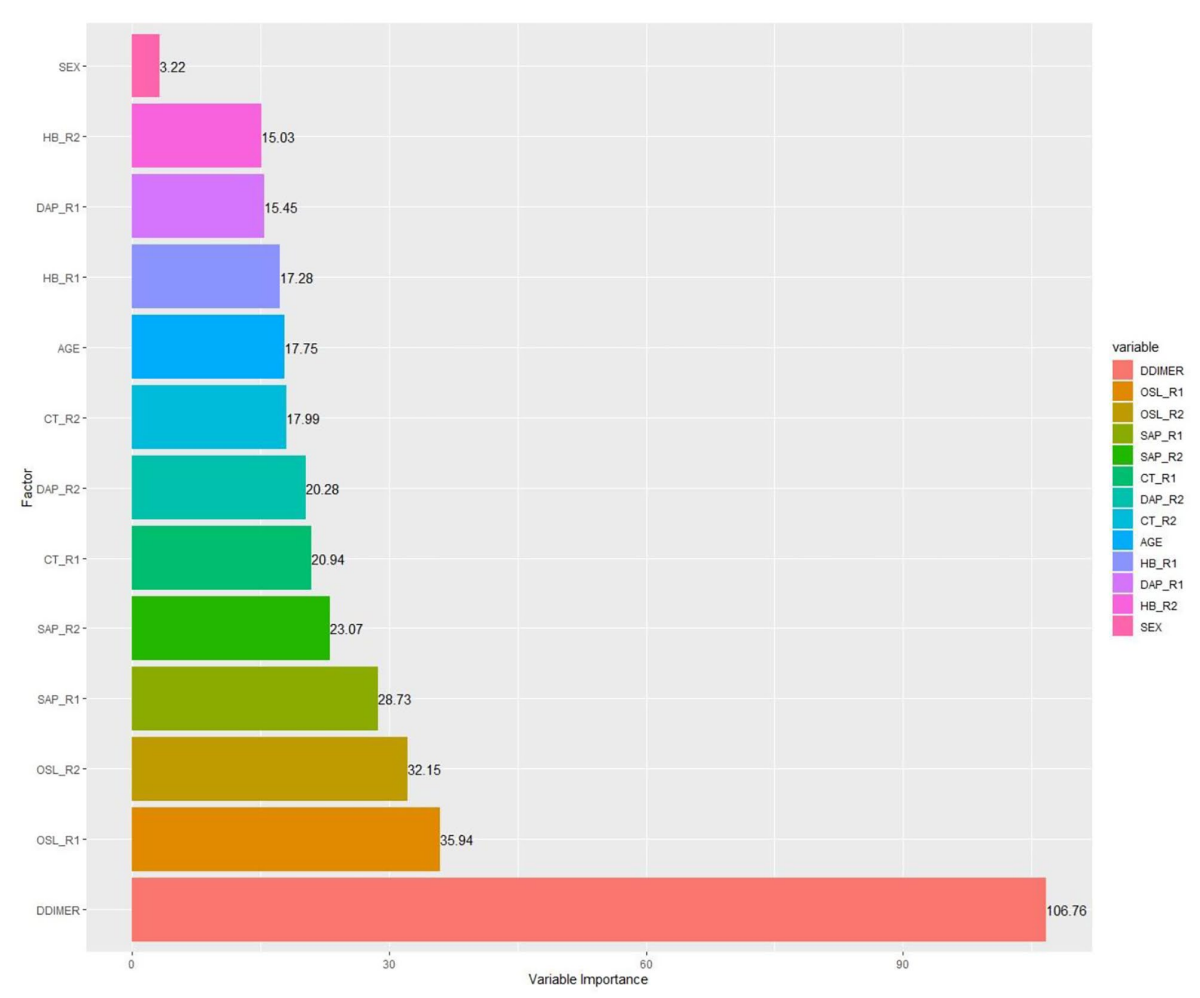
Mean Decrease of Gini Coefficient for candidate predictors

ANOVA tests (α = 0.05) and MDGC (Fig. 2) evidenced the features’ significance. Specifically, the resulting *p*-values (Table 1) were lower than the alpha level (0.05), and the associated factors were therefore categorized as significant for predicting the mechanical ventilation probability. The choice of the features was also underpinned by the MDGC, whose value ranged from 3.22 to 106.76, thereby indicating the high importance of these variables in the RF model. It is good to note the extreme importance of D-DIMMER (MDGC = 106.76) in predicting the outcome variable.

**Fig. 3:**
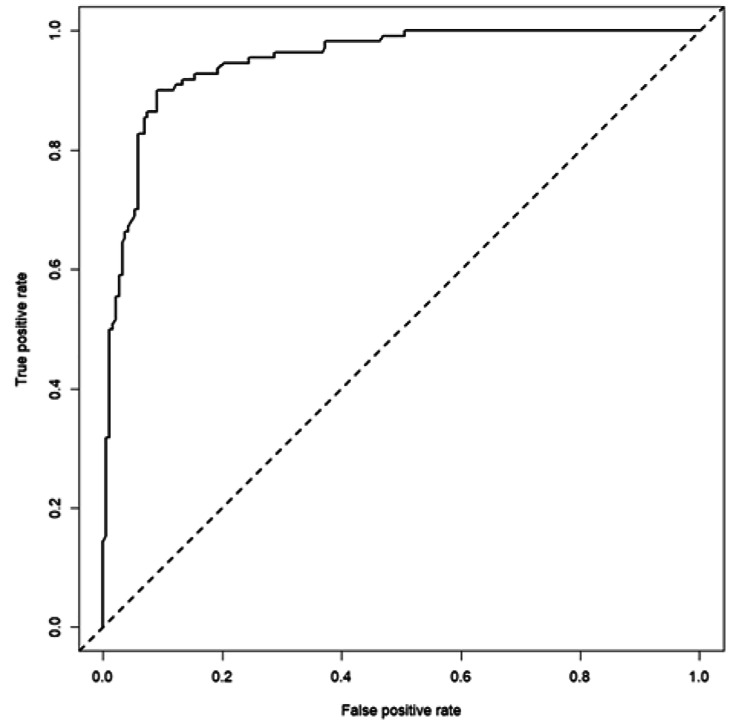
ROC curve for the prediction of mechanical intervention probability in the test subset

Most performance metrics are above 90%, indicating that the RF model can perform well. For example, the sensitivity (93.08 − 95% CI [88.46 − 96.26%]) indicates the true positive rate, i.e., it will correctly predict between 88.46% and 96.26% of the patients requiring ventilation assistance. Meanwhile, the specificity (85.45 − 95% CI [77.45 − 91.45%]) shows the false negative rate, indicating that it will correctly predict 77.45% and 91.45% of cases in which ventilators will not be necessary. Finally, the Receiver Operator Characteristic (ROC) curve (Fig. 3) (95% − 95% CI [89.25 − 100%]) indicates excellent discrimination between patients needing and not needing ventilation support. It is good to highlight that McNemar’s test *p*-value (0.2012) was higher than the error level (0.05), thereby discarding heterogeneity problems between the proportion of miscategorized patients presented in both classes.

**Fig. 4:**
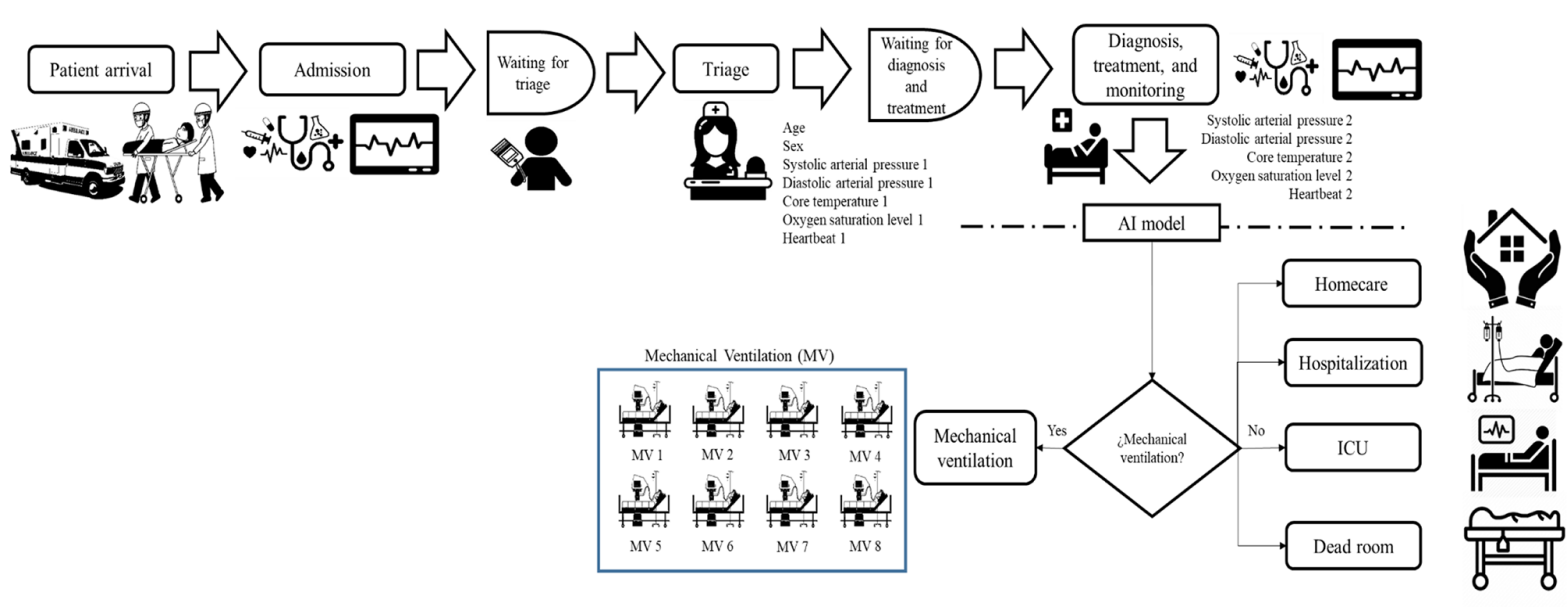
Proposed ED procedure for predicting mechanical ventilation needs based on AI

The RF predictions were then included in the DES model that represents the current ED service during the seasonal respiratory disease (Fig. 4). Although it would have been more useful to implement the AI model earlier at the triage stage, the variables collected in this unit (in the case of the showcased hospital) did not have sufficient predictive power, and it was, therefore, necessary to add other variables gathered in upstream steps of the emergency care. In other words, employing an AI model only based on triage-related indicators would have led to more significant errors in the identification of patients with need of ventilation and capability management decisions.

The flowchart shown in Fig. 4 evidences the multiphase nature of the emergency care. In this service, four main process variables were acknowledged: Time Between Admissions (TBA), Triage Time (TT), ED Length of Stay (ED-LoS), and Mechanical Ventilation Duration (MVD). The mean TBA was 19.06 min (SD: 29.52), while the average TT was 12.5 min. On the other hand, the mean ED-LoS was 52.5 min for I-II triage categories while it was 42.5 min for III-V. The average MVD was found to be 10.1 days. Likewise, the input data analysis concluded that all variables are random (*p* > 0.05). In the case of TBA, different patterns were observed considering the weekday and Time Slot (TS) (TS1: 00:00–08:00; TS2: 08:00–16:00; TS3: 16:00–00:00; *p* = 0) while two groups of patients were evident from the triage process (*p* < 0.05) (I-II; III-V). Chi-squared tests were finally executed to derive each indicator’s probability expression (Table 2).

**Table 2:** Probability expressions of process variables in the DES model

Variable		Pipeline	Probability expression
*TBA*	*Monday – TS1*		LOGN(0.06, 0.15) days
	*Monday – TS2*		LOGN(0.02, 0.04) days
	*Monday – TS3*		LOGN(0.01, 0.02) days
	*Tuesday – TS1*		LOGN(0.09, 0.21) days
	*Tuesday – TS2*		LOGN(0.02, 0.04) days
	*Tuesday – TS3*		LOGN(0.01, 0.02) days
	*Wednesday – TS1*		LOGN(0.14, 0.72) days
	*Wednesday – TS2*		LOGN(0.02, 0.05) days
	*Wednesday – TS3*		LOGN(0.01, 0.02) days
	*Thursday – TS1*		EXPO(0.06) days
	*Thursday – TS2*		LOGN(0.03, 0.06) days
	*Thursday – TS3*		LOGN(0.01, 0.02) days
	*Friday – TS1*		WEIB(0.05, 0.69) days
	*Friday – TS2*		LOGN(0.02, 0.05) days
	*Friday – TS3*		LOGN(0.01, 0.02) days
	*Saturday – TS1*		WEIB(0.04, 0.89) days
	*Saturday – TS2*		LOGN(0.03, 0.06) days
	*Saturday – TS3*		LOGN(0.02, 0.03) days
	*Sunday – TS1*		LOGN(0.05, 0.18) days
	*Sunday – TS2*		LOGN(0.03, 0.05) days
	*Sunday – TS3*		LOGN(0.02, 0.02) days
*TT*		---------	UNIF (10,15) min
*ED-LoS*	*I-II*		UNIF (42.5,62.5) min
	*III-V*		UNIF (32.5,52.5) min
*MVD*	---------		UNIF (7.1, 13.2) days

**Fig. 5:**
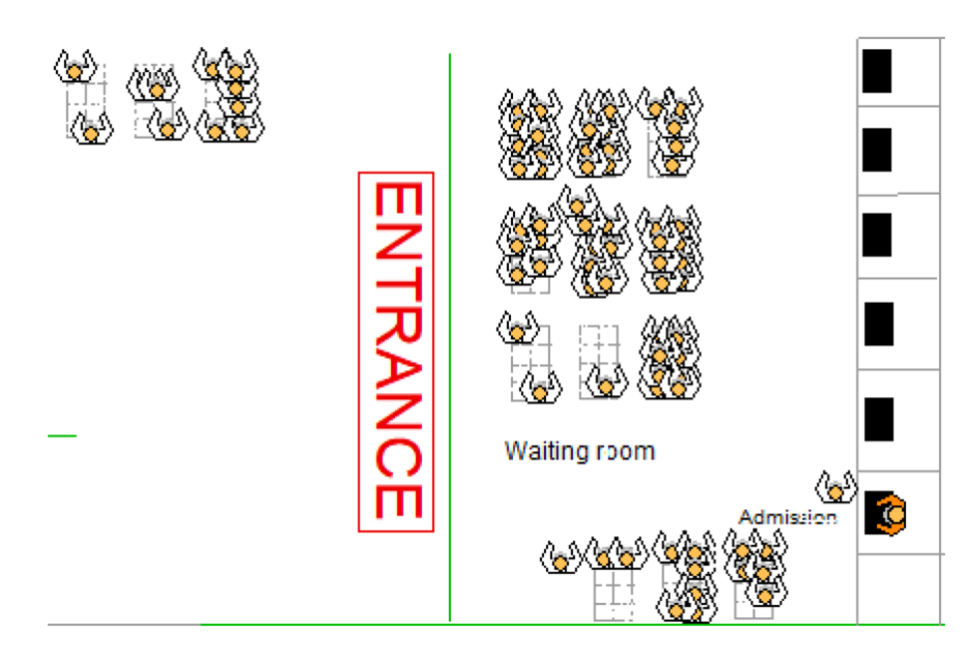
Virtual representation of patient arrival, waiting time before triage, and admission

**Fig. 6:**
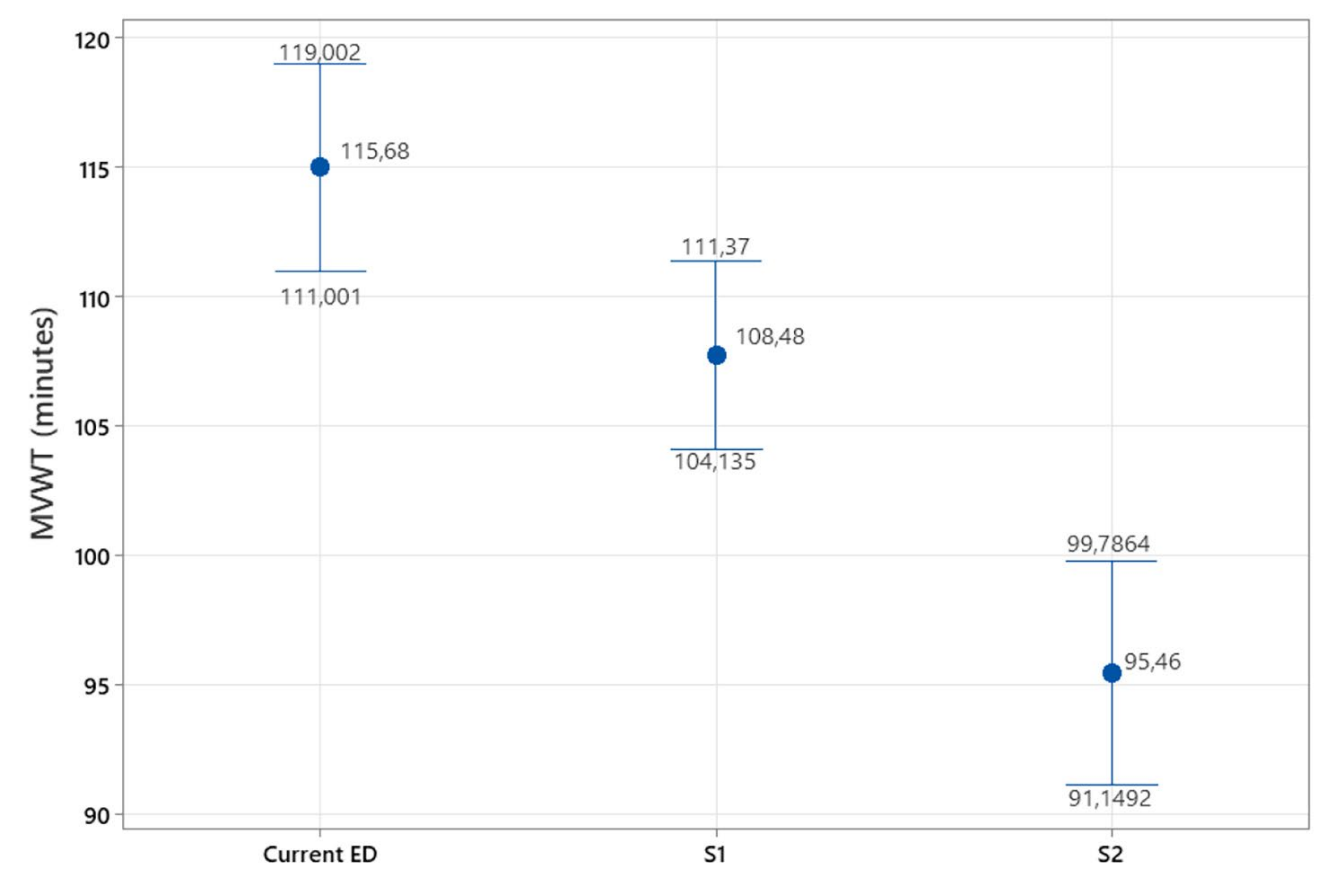
Comparison among the current ED configuration and strategies S1, S2

Figure 5 depicts an ED simulation model compartment designed using Arena®. The iteration length was 15 days (24 h/day), whilst the warm-up period and blocking likelihood were 2400 h and 0, respectively. In this case, 29 iterations were necessary to fully represent the real-world variation of the ED when facing this respiratory viral illness. A 1-sample sign test (α = 0.05) (Ho: η = 116 min|| Ha: η ≠ 116 min; *p*-value = 1) demonstrated that the virtual model is comparable with the real-world ED and can be therefore utilized for operability analysis and pre-testing of interventions aiming at shortening the Mechanical Ventilation Waiting Time (MVWT). Currently, the MVWT ranges from 111.001 to 119.002 min, with a median of 115.68 min. Being aware of this situation, the ED manager and the supervision board proposed two potential improvement strategies: (i) Increase the number of ventilators by 100%, and (ii) Transfer patients to a partner hospital offering idle mechanical ventilation capacity (15 ventilators). We undertook a Mann-Whitney test to determine if the proposed interventions would trigger a substantial MVWT reduction (Fig. 6). In the case of application, Strategy (i) would decrease this indicator between 2.22 and 13.23 min (95% CI; *p*-value = 0.009; W = 930; Improvement percentage = -6.22%) while Strategy (ii) would cause a reduction fluctuating between 11.35 and 20.59 min (95% CI; *p*-value = 0; W = 3,037; Improvement percentage = -17.48%).

## Discussion

Integrating AI and DES in our study offers significant advancements in clinical decision support to emergency care, particularly in mechanical ventilation management during respiratory outbreaks such as the recent COVID-19. This study leverages a novel approach, combining the predictive power of AI with the comprehensive DES process modeling, focusing on improving the mechanical ventilation availability in EDs. The use of RF to estimate the mechanical ventilation probability of respiratory-affected patients at the entry point of emergency wards is a critical component of our approach. The RF predictive accuracy in identifying patients who require mechanical ventilation offers several advantages:

- *Enhanced Triage Accuracy*: By accurately predicting the mechanical ventilation likelihood, EDs can prioritize patients more effectively, ensuring those in need of urgent care receive the earliest attention.
- *Reduction in Medical Errors*: The AI-driven predictions reduce the risk of human error in the triage process, leading to better patient outcomes and reduced mortality rates.
- *Resource Optimization*: The ability to forecast patients in critical respiratory conditions aids in optimal resource allocation, particularly in managing the mechanical ventilator demand.

Our methodology underscores the **interaction between Discrete-Event Analysis** and **emergency care**. By feeding the RF predictions into the DES framework, we can simulate and evaluate the ED response regarding ventilator availability. This interaction provides valuable insights:

- *Scenario Analysis and Planning*: The DES model allows for testing various scenarios, helping hospitals prepare for different ventilator demand levels.
- *Strategic Intervention Development*: The simulation assists in identifying the most effective interventions to alleviate ventilator scarcity, ensuring high availability for critical patients.

Particularly, the DES application offers a detailed understanding of the patient’s journey through the ED. Key benefits include:

- *Process Optimization*: DES helps visualize and analyze the ED processes, from patient admission to mechanical ventilation, highlighting areas for efficiency improvement.
- *Predictive Modeling*: It enables the hospital to foresee and prepare for future mechanical ventilation demands, facilitating better management during high-demand scenarios.

Our findings contribute to the growing research body on using AI and simulation models in healthcare. Applying AI to predicting the mechanical ventilation requirement is consistent with previous studies [2, 6, 10, 11], which have demonstrated the potential of AI in enhancing healthcare delivery. However, our approach goes a step further by integrating these predictions into DES models, offering a more comprehensive tool for ED resource management.

This integrated approach aligns with the current trend toward digital transformation in healthcare, where data-driven and simulation-based strategies are increasingly employed for resource management. Our study serves as a model for other healthcare settings facing similar challenges, especially in resource-constrained environments.

While our study presents a novel approach to managing ED resources during respiratory disease seasons, it has limitations. The accuracy of our AI model is contingent on the data quality and comprehensiveness. The model’s predictions may be less reliable when data collection is inconsistent or incomplete. Additionally, the study’s findings are based on a specific context and healthcare setting, which may limit their generalizability to other settings with different patient demographics or healthcare infrastructures.

Implementing the proposed AI and DES-based framework in a real-world healthcare setting involves several practical and operational challenges. These include ensuring the availability of high-quality data, integrating the model into existing healthcare IT systems, and training healthcare staff to use and interpret the model’s outputs effectively. Addressing these challenges requires a collaborative effort involving clinicians, IT professionals, and healthcare administrators [25].

Definitively, integrating AI with DES in emergency care provides a robust framework for improving decision-making in EDs. It enhances the accuracy of triage processes, optimizes resource allocation, and prepares healthcare systems for efficient mechanical ventilator management, ultimately improving patient outcomes and healthcare efficiency during critical respiratory seasons. This is important considering that a shortage of ventilators may represent a high risk for patient’s health as the ventilators replace the respiratory function during hypoxemic and hypercapnic respiratory failure [26]. This study signifies a substantial step forward in the application of AI and simulation technologies in emergency care. By providing accurate predictions and simulating various scenarios, this approach offers a valuable tool for healthcare providers in making informed decisions about patient care and resource allocation.

### Future developments and implementations

Recognizing the model’s importance in predicting the necessity for mechanical ventilation underscores its potential in healthcare settings. The proposed innovative approach not only boosts the precision in forecasting ventilation needs but also ensures the strategic allocation of ventilators, which are vital during periods when respiratory ailments exert pressure on healthcare resources.

Table 3 outlines the practical utility of the decision support tool in a clinical setting.

**Table 3:** Practical utility of the decision support tool

Utility Aspect	Description
Real-time Monitoring and Alerts	Continuously analyzes incoming patient data to identify those at high risk of requiring mechanical ventilation and alerts medical staff for timely intervention.
Resource Allocation	Predicting ventilation needs is crucial during peak demand periods and aids in the efficient allocation of ventilators and ICU beds.
Training and Education	It serves as an educational resource for medical staff, especially those in training, by providing insights into the predictive factors for respiratory support needs.
Data-Driven Decisions	Facilitates a more data-driven approach to patient care, reducing variability in clinical judgment and potentially leading to more standardized care pathways.
Integration with Electronic Health Records (EHRs)	Integrates with EHRs to leverage historical patient data for more accurate predictions and contributes to a comprehensive patient care record.

Future developments will enhance the model by incorporating a broader range of clinical parameters and patient data, aiming to boost predictive accuracy and extend its applicability across various patient demographics. Collaboration with clinical entities for real-world testing and refinement of the model is anticipated, solidifying its relevance in clinical decision-making processes.

The model’s utility as a decision support mechanism is highlighted by its capability to objectively assess clinical situations, reducing reliance on subjective evaluations. This is particularly advantageous in high-demand scenarios or when the medical team’s experience varies, ensuring that patients most at risk of requiring mechanical ventilation are identified promptly. Consequently, this facilitates the prioritization of patient care, efficient resource utilization, and improved patient outcomes.

Explorations into integrating the predictive tool within hospital information systems are underway. These aim to offer real-time support for clinical decisions. Such integration is expected to enhance workflow efficiency, enabling early identification and proactive management of high-risk patients.

The development and implementation of this predictive model are critical steps forward in improving the delivery of emergency care, especially during peak times. The ongoing refinement and integration into clinical workflows are intended to establish a robust decision support system, enhancing care quality and efficiency for patients potentially needing mechanical ventilation.

Of course, it is important to point out that enhancing the predictive algorithm for mechanical ventilation requires a strategic approach centered on key elements. First, broadening the dataset with diverse clinical parameters and patient demographics is crucial for improving model generalizability. Adopting advanced machine learning techniques like deep learning will enable the model to uncover complex data patterns. New real-world validation through collaboration with healthcare institutions ensures the algorithm’s effectiveness in practical settings. Implementing a continuous learning feedback loop will allow the model to evolve by integrating new patient data and outcomes. Lastly, interdisciplinary collaboration will ensure the model’s clinical relevance and seamless integration with healthcare IT systems, making it a robust tool for clinical decision-making.

## Conclusions

The context of seasonal respiratory diseases, including the recent pandemic and the challenges regarding mechanical ventilation management in EDs, motivate the need for more agile and robust methodological approaches to increase capacity due to the expected demand load and the flexibility and ease for timely mechanical ventilator allocation. In this study, we proposed integrating AI and DES to improve the mechanical ventilation availability in EDs during respiratory disease seasons. This approach is helpful for ED managers as it provides decision-making support for enhanced triage accuracy, reduced medical errors, scenario analysis and planning, and strategic intervention development.

An important aspect of caring for such critically ill patients, with acute respiratory failure, is the additional need for providing diverse human and technical assistance for ancillary activities such as: patient anaesthetization, tubing, and sedation, necessitating additional medical and nursing support. Once ventilator requirements are determined, further work should focus on determining the necessary human and other equipment to provide such additional resources, which may be in short supply. In this regard, the DES model may evaluate how the availability of staff and medical supplies may be managed to respond to mechanical ventilation demands during respiratory disease seasons effectively [27, 28].

Digital Twins have recently emerged as a powerful tool for providing a virtual representation of real-life systems and continuously updating with real-time data; simultaneously, the twin is permitted to interact with and improve the live systems. Such Digital Twinning is a powerful approach for managing hospital systems in resource-limited settings and then represents a natural extension of our current models. Another useful direction for further work is extending our approach to resourcing other ED areas. Likewise, it is widely advised to collect other variables in the triage stage, including those associated with the respiratory rate and blood gas analysis, to move the model to the beginning of the emergency care and consequently intervene more anticipatively in the high-risk patients and the mechanical ventilation capacity. These ideas could readily be extended to the development of flexible and generalizable healthcare resource modeling, even linked to readmissions from the community.

## Data Availability

All the data are available for review upon request.

## Abbreviations

AI: Artificial Intelligence
ANOVA: Analysis of Variance
CI: Confidence Interval
DES: Discrete-Event Simulation
ED: Emergency Department
ED-LoS: Emergency Department Length of Stay
EHR: Electronic Health Record
IT: Information Technology
MDGC: Mean Decrease in Gini Coefficient
MVD: Mechanical Ventilation Duration
MVWT: Mechanical Ventilation Waiting Time
OSL: Oxygen Saturation Level
RF: Random Forest
ROC: Receiver Operator Characteristic
RSV: Respiratory Syncytial Virus
TBA: Time Between Admissions
TS: Time Slot
TT: Triage Time
UK: United Kingdom
